# Wine fermentation microbiome: a landscape from different Portuguese wine appellations

**DOI:** 10.3389/fmicb.2015.00905

**Published:** 2015-09-01

**Authors:** Cátia Pinto, Diogo Pinho, Remy Cardoso, Valéria Custódio, Joana Fernandes, Susana Sousa, Miguel Pinheiro, Conceição Egas, Ana C. Gomes

**Affiliations:** ^1^Genomics Unit, Biocant – Biotechnology Innovation Center, CantanhedePortugal; ^2^GenoInSeq Unit, Biocant – Biotechnology Innovation Center, CantanhedePortugal

**Keywords:** grape microbiology, wine spontaneous fermentation microbiome, industrial metagenomics

## Abstract

Grapes and wine musts harbor a complex microbiome, which plays a crucial role in wine fermentation as it impacts on wine flavour and, consequently, on its final quality and value. Unveiling the microbiome and its dynamics, and understanding the ecological factors that explain such biodiversity, has been a challenge to oenology. In this work, we tackle this using a metagenomics approach to describe the natural microbial communities, both fungal and bacterial microorganisms, associated with spontaneous wine fermentations. For this, the wine microbiome, from six Portuguese wine appellations, was fully characterized as regards to three stages of fermentation – Initial Musts (IM), and Start and End of alcoholic fermentations (SF and EF, respectively). The wine fermentation process revealed a higher impact on fungal populations when compared with bacterial communities, and the fermentation evolution clearly caused a loss of the environmental microorganisms. Furthermore, significant differences (*p* < 0.05) were found in the fungal populations between IM, SF, and EF, and in the bacterial population between IM and SF. Fungal communities were characterized by either the presence of environmental microorganisms and phytopathogens in the IM, or yeasts associated with alcoholic fermentations in wine must samples as *Saccharomyces* and non-*Saccharomyces* yeasts (as *Lachancea, Metschnikowia, Hanseniaspora, Hyphopichia, Sporothrix, Candida*, and *Schizosaccharomyces*). Among bacterial communities, the most abundant family was Enterobacteriaceae; though families of species associated with the production of lactic acid (Lactobacillaceae, Leuconostocaceae) and acetic acid (Acetobacteriaceae) were also detected. Interestingly, a biogeographical correlation for both fungal and bacterial communities was identified between wine appellations at IM suggesting that each wine region contains specific and embedded microbial communities which may contribute to the uniqueness of regional wines.

## Introduction

The knowledge and the understanding of the microbial *terroir* – how the microbiome contributes to the natural environment of grapes and to the identity of wine, is a process that starts at the vineyards, at the harvest of grapes, and then evolves along the different stages of fermentation (Van Leeuwen and Seguin, 2006; Bokulich et al., 2013). Indeed, it is known that grapes harbor a complex microbiome, including a high range of filamentous fungi, yeasts and bacteria with different physiological and metabolic characteristics (Pretorius, 2000; Fleet, 2003; Barata et al., 2012). The microflora of the grapes is highly variable, mostly due to the influence of external factors as environmental parameters, geographical location, grape cultivars and application of phytochemicals on the vineyards (Pretorius, 2000; Cadez et al., 2010; Pinto et al., 2014). These microbial communities play an important role during the winemaking process, as they metabolize the sugars from the grapes and produce a whole set of secondary metabolites that influence the wine aromatic quality (Fleet, 2003). In fact, the natural diversity of those metabolic pathways, and the contribution of the different microorganisms involved on the fermentation process, is well documented (Setati et al., 2012). Therefore, unveiling the microbial biodiversity of grapes and during their fermentation will expand our understanding on fermentation dynamics, on its control (Bisson, 1999; Bisson and Butzke, 2000) and may also contribute to the identification of novel starter cultures (Fleet, 2008; Ciani et al., 2010).

The spontaneous wine fermentation is carried out by indigenous microbiota (Heard, 1999; Pretorius, 2000; Ciani et al., 2006; Renouf et al., 2007). Species of *Metschnikowia, Candida, Hanseniaspora*, *Pichia, Lachancea* (*Kluyveromyces*), and *Saccharomyces* are often present at the initial stages of wine fermentations and form the dominant consortium (Cocolin et al., 2000; Mills et al., 2002; Fleet, 2008). However, during the wine fermentation, the ethanol content increases and *Saccharomyces cerevisiae* strains dominate the alcoholic fermentation (AF; Fleet, 2008). Additionally, a deacidification may occur, by conversion of malic acid into lactic acid. This process is known as malolactic fermentation (MLF) and is due to the activity of lactic acid bacteria (LAB; Lonvaud-Funel, 1999; Lerm et al., 2011). The LAB species associated with MLF generally belong to the *Oenococcus, Pediococcus, Lactobacillus*, and *Leuconostoc* genera (Lonvaud-Funel, 1999). Indeed, MLF mainly influences the organoleptic characteristics and the aging of wines (Lonvaud-Funel, 1999). On the other hand, acetic acid bacteria (AAB) may cause a negative impact on the winemaking process, due to the production of undesirable metabolites, as acetic acid, thus affect negatively the quality of wine and so are considered spoilage microorganisms (Zoecklein et al., 2000).

The majority of the wine microbiology studies focus on the characterization of *S. cerevisiae* strains (Pretorius, 2000; Fleet, 2008; Nisiotou et al., 2011). Nevertheless, recent studies based on culture-independent methods, started to explore the microbial communities associated with wine grapes (Bokulich et al., 2013; Taylor et al., 2014). It is widely accepted that unveiling the indigenous microbial community associated with particular grape varieties, from specific locations, could represent an important source of distinctive metabolites and introduce an authenticity *terroir* to the region (Heard, 1999; Jolly et al., 2006; Fleet, 2008). The biogeographical distribution of the wine associated microorganisms has been recently investigated in vineyards from different regions of California (Bokulich et al., 2013), New Zealand (Taylor et al., 2014), and in conventional, biodynamic, and integrated vineyards of South Africa (Setati et al., 2012). These studies allowed for a better spatial and temporal characterization of the wine grapes microbiome and brought new insights of its dynamics and biodiversity. Also, other biogeography wine studies have been previously published focusing on *S. cerevisiae* (Schuller et al., 2012). Nevertheless, there is still a lack of knowledge on the diversity and the dynamics of microbial communities as a whole– from the wine grapes until the wine fermentation, which can now be obtained using high-throughput sequencing technologies and metagenomics approaches that allow for the identification of both non-cultivable microorganisms, and of less represented species.

In this work, a total of six different Portuguese wine appellations were considered to analysis and high-throughput sequencing was used to unveil the wine microbiome present at initial musts (IM), and start and end of alcoholic fermentations (SF and EF, respectively). This work aims to understand the dynamics of microbial communities across spontaneous wine fermentations and also to reveal the biogeographic distribution of grape and wine microbiomes of Portuguese wine appellations.

## Materials and Methods

### Grape Sampling, Laboratory-Scale Fermentation, and DNA Extraction

The grape samples were collected during the 2010 vintage, from six different Portuguese appellations, namely, Minho (Mi), Douro (Dr), Dão (D), Bairrada (B), Estremadura (E), and Alentejo (Al). For each appellation, the three most representative grape varieties were considered for sampling, with exception of Minho where only two grape varieties were considered (**Supplementary FigureS1**). For all regions, the sampling was carried out 1 day prior the harvest. The sampling was authorized by private wine producers, who are fully acknowledged in this paper, and no specific permissions were required for this activity. Also, the field study did not involve any endangered or protected species.

For each appellation, one vineyard (farm) with different grape varieties was selected, and for each grape variety, 2 kg of healthy and undamaged grapes were collected. Grapes were collected from multiple bunches of different grapevines, randomly distributed across the vineyard in order to assure the representativeness of the sampling. These samples were collected into sterile plastic bags and transported to the laboratory chilled on ice. In total, 17 grape samples were collected, crushed and allowed for laboratory-scale fermentation (spontaneous AF) under aseptic conditions and acclimatised at 21°C, at the Genomics Unit from Biocant. For each sample, the microbial diversity was analyzed at three stages: IM, corresponding to the juice of crushed grapes; start of alcoholic fermentation (SF) and end of alcoholic fermentation (EF), which corresponded to the weight loss of 5 and 70 g/L of sugar, respectively. The SF and EF where daily monitored through weighting. At each stage, 50 mL of wine must were collected and centrifuged at 4000 rpm for 10 min. The respective microbial pellets were collected, washed twice with 0.9% NaCl and re-suspended with glycerol. A total of 51 samples (*n* = 17 × 3 fermentation stages) were stored at -80°C for DNA extraction. The DNA from each individual sample was extracted using the DNeasy Plant mini kit (QIAGEN, USA), according to the manufacturer’s instructions, with a prior cell rupture using glass beads in Tissue Lyser (Qiagen, USA), to assure full disruption of microbial cells.

### rDNA Library Construction and Pyrosequencing

A PCR amplicon library was built for each individual sample. For a better discrimination of the entire microbial community present during the fermentation process, rDNA sequences from both prokaryotic and eukaryotic microorganisms were amplified, using PCR primers that were designed to target three distinct regions. The V6 hypervariable region of the 16S rRNA was used for the identification of prokaryotic microorganisms (Sogin et al., 2006) and the D2, from the 26S rRNA, and ITS2 regions (White et al., 1990) for eukaryotic identification. The sequence-specific portions of the used primers were: V6_F 5′-ATGCAACGCGAAGAACCT-3′ and V6_R 5′-TAGCGATTCCGACTTCA-3′ of V6 region; D2_F 5′-AAGMACTTTGRAAAGAGAG-3′ and D2_R 5′-GGTCCGTGTTTCAAGACG-3′ of D2 region; and ITS2_F 5′-GCATCGATGAAGAACGC-3′ and ITS2_R 5′-CCTCCGCTTATTGATATGC-3′ of ITS2 region. Additionally, the fusion primers also contained a specific Roche 454 adaptor sequence and a multiplex identifier sequence with eight nucleotides, which allows the pooling of amplicons.

All PCR reactions were carried out in 30 μL reaction mix containing 2 μL of DNA template, 1.5 units of FastStart High Fidelity Taq DNA polymerase (Roche, USA), 1x reaction buffer with MgCl_2_ (1.8 mM) incorporate (Roche, USA), 0.2 mM dNTPs (Bioron, Germany) and 0.8 μM of the forward and reverse primers for V6 region or 0.4 μM of forward and reverse primers for D2 and ITS2 regions. For prokaryotes amplification, cycling conditions consisted in a first denaturation step at 94°C for 5 min followed by 20 cycles with a denaturation step at 94°C for 35 s, annealing at 50°C for 35 s and an extension at 72°C for 40 s. A final extension cycle at 72°C for 5 min was applied. The cycling conditions applied for eukaryotic microorganisms were the same, but the PCR consisted in 25 cycles. The amplification success was assessed by electrophoresis using the HT DNA 5K/RNA LabChip for the LabChip 90 (Caliper Life Sciences, USA). The PCR reaction products were then purified with the High Pure 96 UF Cleanup Plates (Roche, USA) and quantified using the PicoGreen^®^ dsDNA quantitation kit (Invitrogen, USA). Samples were pooled together according to the number of DNA molecules, in equimolar concentrations and submitted for pyrosequencing using the GS FLX Titanium platform (454 Life Sciences, Roche) at Biocant, Portugal. The raw data obtained was deposited in NCBI platform with the accession number SRA097159.

### Bioinformatic Data Analysis

Raw sequence reads were processed with MetaBiodiverse, an automatic annotation pipeline fully implemented at Genoinseq of Biocant (Vaz-Moreira et al., 2011; Egas et al., 2012; Pinto et al., 2014). Briefly, the raw data obtained was split through the identification of barcode sequences and quality filters were applied to remove low quality reads. Thus, (*i*) short sequences (<120 bp), (*ii*) sequences containing more than two undetermined nucleotides (N), (*iii*) masked sequences with more 50% of low complexity areas (Sogin et al., 2006) and (*iv*) chimera sequences, detected using UChime were removed (Edgar et al., 2011). All sequences with a distance value below 0.03, which corresponds to the species-level threshold (Sharpton et al., 2011), were grouped in operational taxonomic units (OTUs) through USearch, version 6.0.307 (Edgar, 2010). The Mothur package (Schloss et al., 2009) was used to generate rarefaction curves (richness of population analysis) and to calculate the population diversity analysis estimator Chao1 (α diversity). For the taxonomic annotation, each generated consensus sequences were queried by BLAST on curated databases. The Ribosomal Database Project II (RDP; Cole et al., 2009) was used for prokaryotic microorganisms assignment and the nt@ncbi/SILVA database for eukaryotic classification. After BLAST, the best hits were selected and subjected to another quality control. All sequences with an alignment of less than 40% or with an *E*-value greater than 1e^-50^ were rejected. Sequences that passed the quality check were subjected to a bootstrap test with 100 replicates, using the seqBoot application from the Phylip package (Felsenstein, 1989). The OTU identification process implemented provided a high level of confidence in taxon assignment of each sequence. The process assessed the correct *E*-values scores, went through the taxonomy path and identified the lowest common taxonomy level in the bootstrap process. Only those sequences with an identity greater than 70% were reported, while all the others went up the taxonomy levels until reached 70%.

### Statistical Analyses

To determine the minimum significant difference (*p* < 0.05) in the biodiversity (Chao1) of IM, SF and EF samples, one-way analysis of variance (ANOVA) was performed using SPSS 20.0 (IBM, US). Shapiro-Wilk normality tests were carried out for each eukaryotic and prokaryotic phylogenetic group. As most groups did not follow the normal distribution, Friedman and Sign tests (pairwise comparisons) were used. The microbial communities were compared at family level for prokaryotic population and at genus level for eukaryotic population through the sequence reads analysis. Thus, microbial population comparisons were carried out using these taxa.

Sequence reads data matrixes of the 97% similarity grouped bacterial and fungal OTUs, produced by Metabiodiverse, were normalized by the total reads obtained for each analyzed sample, and then log(X+1) transformed and used to calculate a Bray–Curtis resemblance matrixes. The data obtained for the three fermentation stages were (i) explored by principal coordinate analysis (PCO), (ii) tested by Analysis of Similarities (ANOSIM) for significant differences and (iii) analyzed by SIMPER to identify the taxa responsible for similarity between samples within each group and dissimilarities between groups, using Primer E software version 6 (Clarke and Gorley, 2006). The same analyses were performed to explore and test the influence of wine appellations on microbiome although, for each fermentation stage, individual matrixes were created in order to remove the “fermentation stage” variable.

## Results

### Diversity and Richness of Microbial Communities

In this study, we assessed and compared the microbial community of IM, and the Start and End of wine alcoholic fermentations (SF and EF, respectively), from six Portuguese appellations by DNA massive parallel sequencing of 16S rDNA for bacteria, and both, ITS2 and D2 for fungal analysis. Two target regions were used for the fungal population identification as previous experiments demonstrated that these combination would allow for the highest coverage of eukaryotic organisms (Pinto et al., 2014).

The deep sequencing of microbial communities generated a total of 1,180,106 sequences of ITS2, D2, and V6 regions from IM, SF, and EF (**Table 1** and **Supplementary Table S1**). A total of 1,160,482 sequences passed the quality control parameters, representing an acceptance of 98.3% of high quality sequences (723,474 eukaryotic sequences: 313,919 reads for ITS2 region and 409,555 for D2 region; and 437,008 prokaryotic sequences). The clustering of the sequences at a phylogenetic distance of 3% generated a total of 1,034 OTUs for ITS2, 1,099 for D2, and 1,461 for V6. The number of OTUs from both eukaryotic and prokaryotic communities decreased along the fermentation.

**Table 1:** Total sequences obtained for eukaryotic (ITS2 and D2) and prokaryotic (V6) microbial community for IM, SF, and EF samples.

		No. Reads		0.03 distance		
Sampling point	Target region	Total	High quality	OTU obtained (mean ± SEM)	Estimated species (mean ± SEM)	Coverage (mean ± SEM)
IM	ITS2	119876	116064	68 ± 6	100 ± 9	68.83 ± 2.26%
	D2	131837	129652	71 ± 6	110 ± 10	66.54 ± 2.52%
	V6	145796	145051	78 ± 12	134 ± 21	60.30 ± 3.19%
SF	ITS2	114993	111075	33 ± 3	47 ± 5	74.44 ± 3.62%
	D2	145559	143100	36 ± 3	56 ± 7	68.63 ± 3.29%
	V6	159940	159054	56 ± 9	83 ± 13	66.92 ± 3.28%
EF	ITS2	90207	86780	20 ± 1	29 ± 4	77.74 ± 4.10%
	D2	138156	136803	19 ± 2	25 ± 2	79.82 ± 3.23%
	V6	133742	132903	54 ± 9	81 ± 12	68.15 ± 3.48%
	Eukaryotic	740628	723474			
	Prokaryotic	439478	437008			
	**Total**	**1180106**	**1160482**			

*Operational taxonomic units (OTUs) and estimated species (chao1) were determined at a genetic distance of 3% using Mothur. The coverage obtained was also determined as being the ratio between the observed OTUs and estimated Chao1 (OTUs/Chao1). A detailed table with indication of the samples origin is provided as **Supplementary Table S1.***

The diversity of microbial community was compared by rarefaction curve analysis (**Supplementary Figure S2**) and the ratio between the number of the obtained and the expected OTUs (predicted by Chao1) was used to determine the coverage for the microbial communities: it was of 73.7 ± 2.0% for ITS2 region, 71.7 ± 1.9% for D2 region and 65.1 ± 1.9% for V6 region (**Supplementary Table S1**).

**FIGURE 1:**
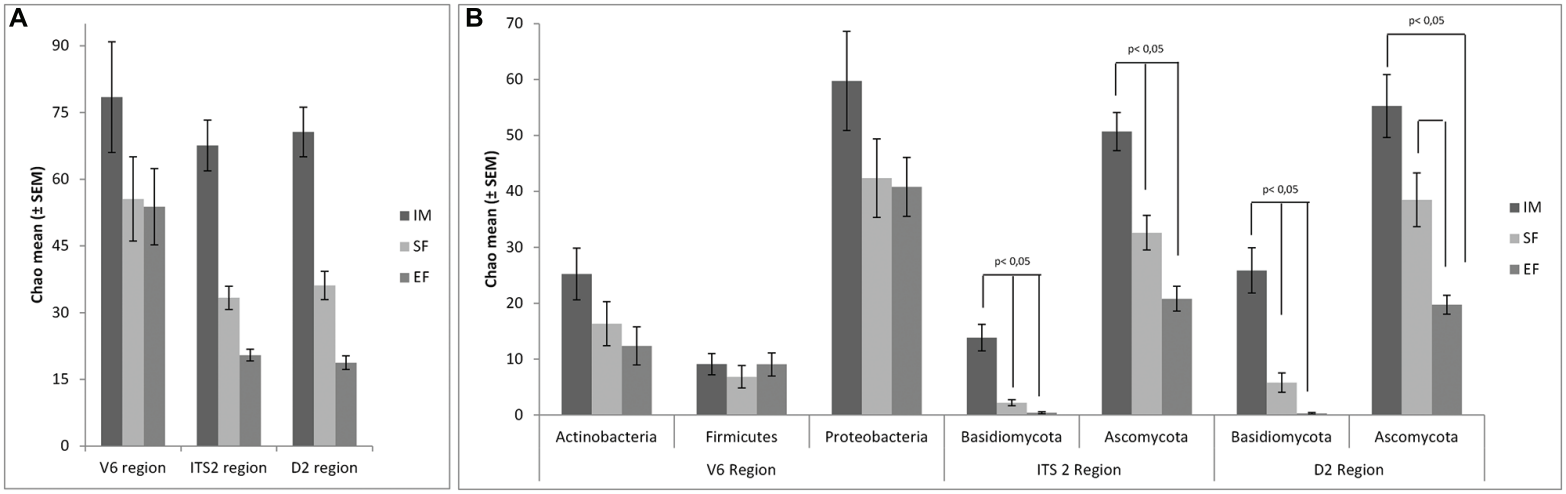
**Biodiversity dynamics associated with V6, ITS2, and D2 region, at domain **(A)** and phylum level (B).** The mean of Chao1 index ± SEM are represented in the graph. Significance was assessed with Friedman test and signal test. *p* < 0.05 was set as statistic significant level.

In order to assess the variations of microbial biodiversity, the Chao1 richness estimator was used to compare the three fermentation stages at both domain and phylum levels. In general, and as expected, a decrease of richness was observed over the spontaneous wine fermentation for both fungi and bacteria, at the analyzed taxonomical levels (domain and phylum; **Figure 1**). Considering the domain (**Figure 1A**), no significant differences were found for the three rDNA regions. At the phylum level, significant differences (*p* < 0.05) in the Basidiomycota between all stages of fermentation were observed (both for ITS2 and D2 regions), and in the Ascomycota population differences were between SF and EF, but not between IM and SF (**Figure 1B**). For the bacterial population, a decrease in biodiversity was observed but no significant differences were detected (V6 rDNA region). A clear relationship was observed between the microbial community biodiversity and the stage of fermentation. Interestingly, the variations of biodiversity, which were observed along the fermentation stages, revealed a higher impact on the structure of the eukaryotic population, when compared with the prokaryotic communities. Moreover, regarding the microbial biodiversity, the prokaryotic population was richer than the eukaryotic population.

### General Characterization of Microbial Communities

**FIGURE 2:**
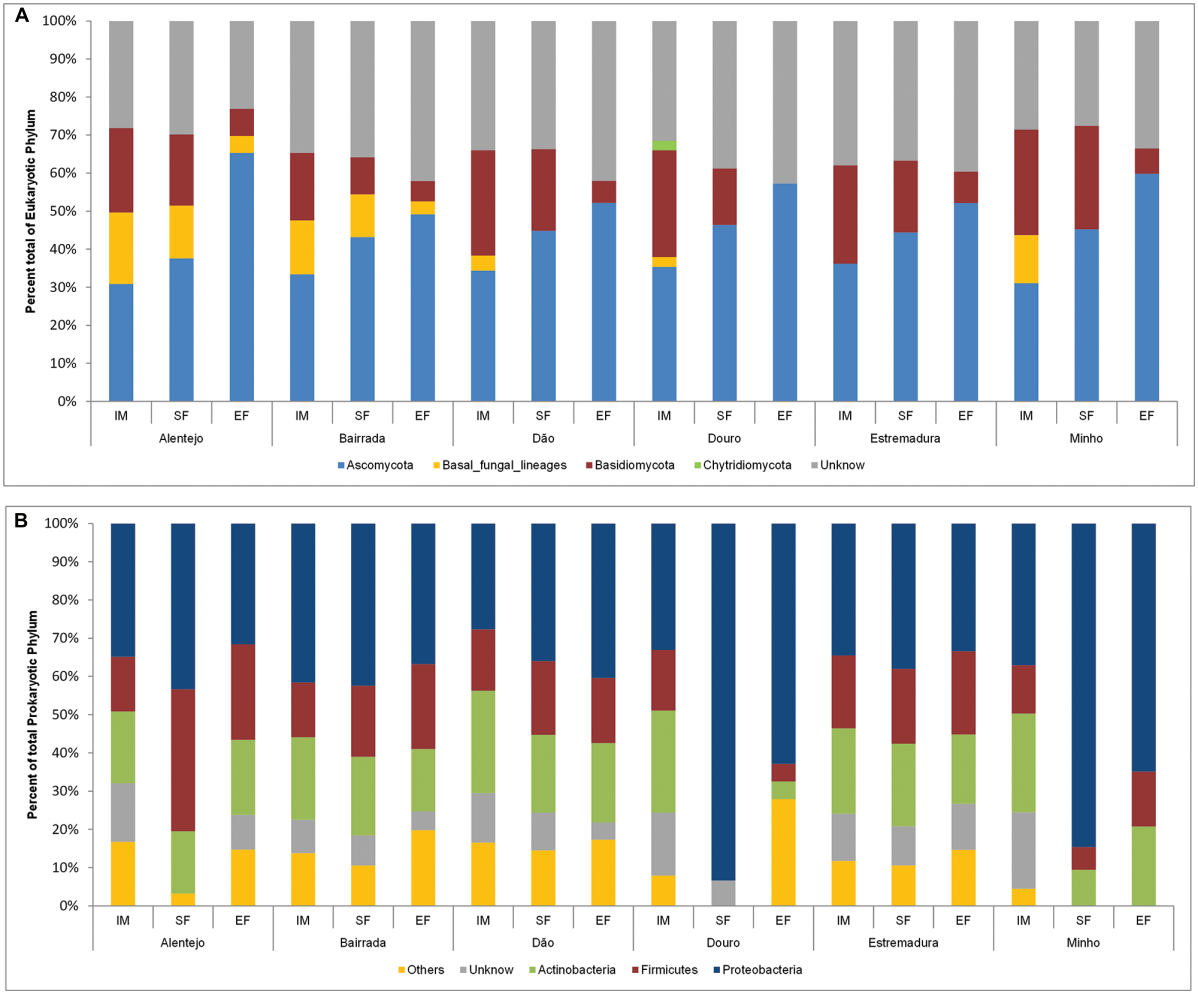
**Eukaryotic **(A)** and prokaryotic **(B)** community distribution over IM, SF, and EF from Portuguese appellations at the phylum level**. Relative abundance of the eukaryotic **(A)** and prokaryotic **(B)** community through phylum analysis. For the whole figure, “Unknown” represents unclassified sequences. The prokaryotic members of rare population phyla were placed in an artificial group designed as “Others” and included Acidobacteria, Bacteroidetes, Chloroflexi, Cyanobacteria, Deinococcus-Thermus, Gemmatimonadetes, Nitrospirae, Planctomycetes, Tenericutes, and Verrumicrobia.

The dominant phylum across the entire eukaryotic population was Ascomycota (42.4%), though it also contained Basidiomycota (17.7%), and other fungi, as Chytridiomycota phylum (0.2%) and *basal fungal lineages* (5.6%). Also, a considerable number of unidentified microorganisms (34.1%) were mostly present at IM (**Figure 2A**).

**FIGURE 3:**
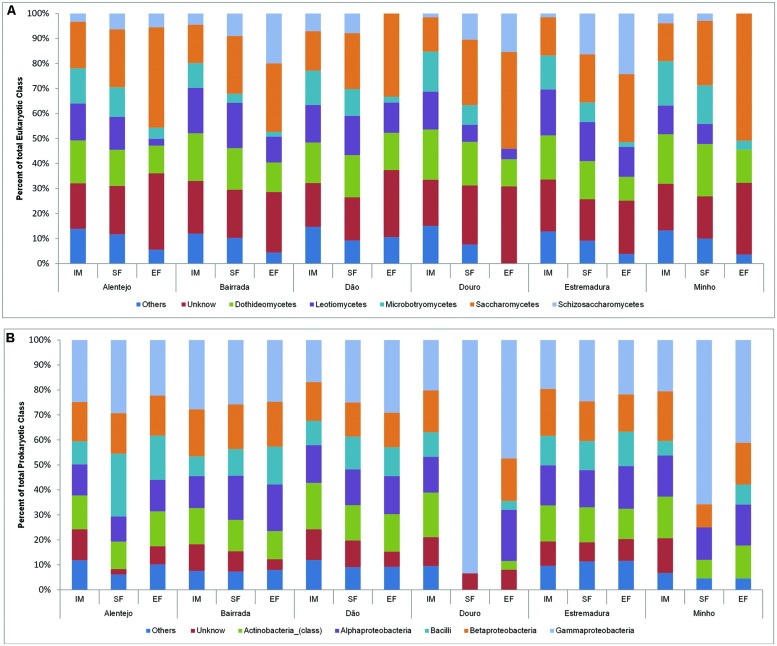
**Eukaryotic **(A)** and prokaryotic **(B)** community distribution over IM, SF, and EF from Portuguese appellations at the class level.** Relative abundance of the eukaryotic **(A)** and prokaryotic **(B)** community through the class analysis. The members of rare population phyla were placed in an artificial group designed as “Others.”

In all samples, the dynamics of microbial populations at phylum level were very similar. Nevertheless, the relative abundances varied along the fermentation and across Portuguese appellations (**Figure 2A**). Microorganisms belonging to Basidiomycota phylum decreased during the fermentation process. To better understand such population dynamics, the relative abundance at class level was analyzed. The entire microbial community was mostly characterized by Saccharomycetes (22.9%), Dothideomycetes (16.2%), Leotiomycetes (12.9%), Microbotryomycetes (9.6%), and Schizosaccharomycetes (7.7%; **Figure 3A**).

Concerning the prokaryotic communities, the dominant phyla were Proteobacteria (41.6%), Actinobacteria (19.2%), and Firmicutes (17.9%; **Figure 2B**). The members of under-represented phyla were grouped together in the artificial group “Other” (12.4%) and included Acidobacteria, Bacteroidetes, Chloroflexi, Cyanobacteria, Deinococcus-Thermus, Gemmatimonadetes, Nitrospirae, Planctomycetes, Tenericutes, and Verrumicrobia. As a reflection of the microbial community dynamics, and as seen in eukaryotic microorganisms, the relative abundances of all prokaryotic communities varied in both time and space. Along the spontaneous wine fermentations, it was possible to observe an increase of microorganisms belonging to the Proteobacteria phylum (**Figure 2B**), thus indicating that samples are losing their environmental characteristics. Regarding the prokaryotic classes, microorganisms from Gammaproteobacteria (27.9%), Betaproteobacteria (15.9%), Alphaproteobacteria (14.8%), Actinobacteria (13.2%), and Bacilli (11.5%) were identified (**Figure 3B**).

### The Landscape of Microbial Communities Throughout Wine Fermentation

**FIGURE 4:**
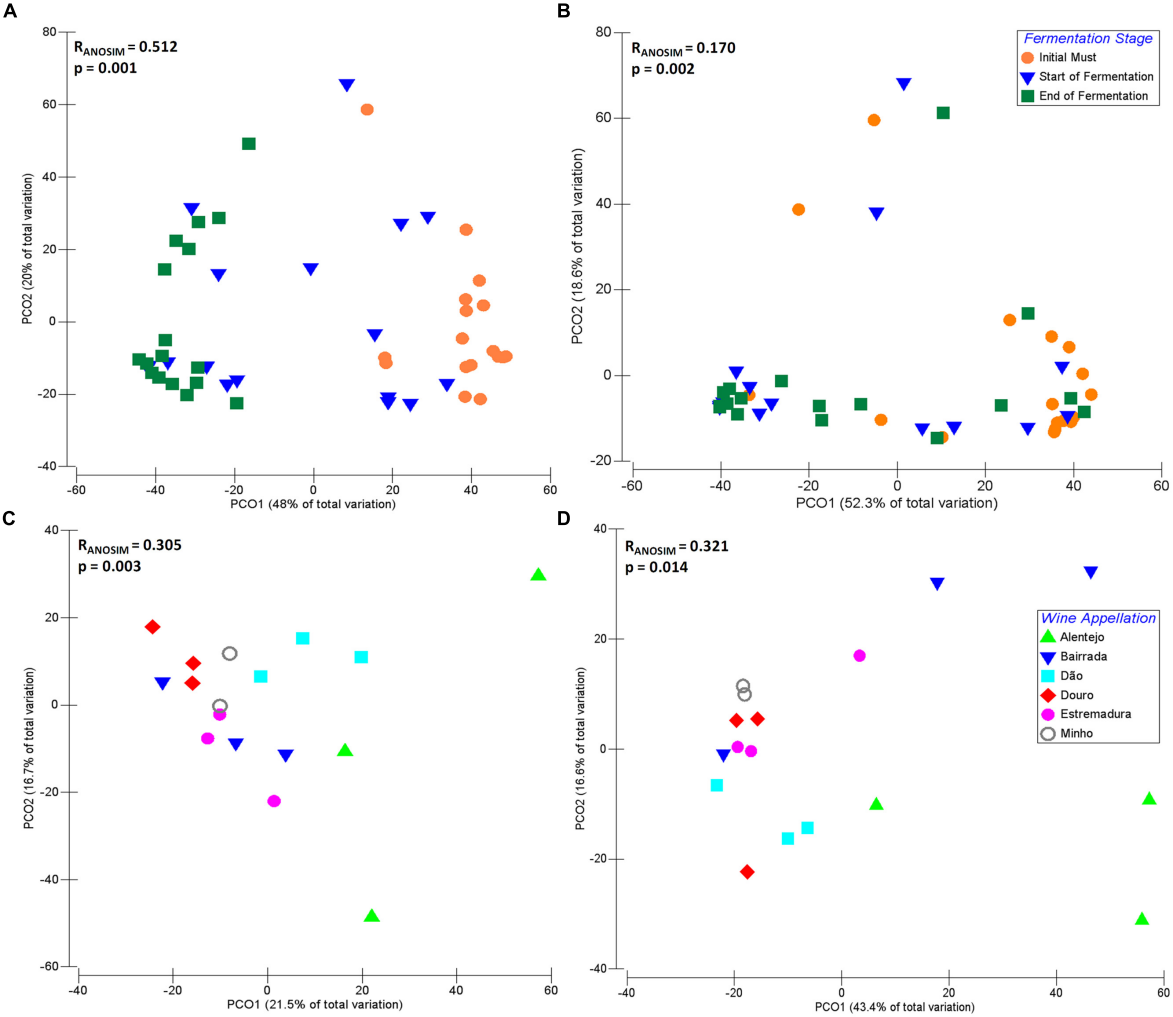
**Principal coordinate analysis (PCO) biplot diagram of microbial community during fermentation process, based on sequence abundance of eukaryotic genus and bacterial family**. Principal coordinates analysis (showing the first and second components) of fungal **(A)** and bacterial **(B)** communities across the fermentation stage namely, initial musts (IM), start of fermentation (SF) and end of fermentation (EF) for Portuguese appellations. Biogeographical distribution of fungal **(C)** and bacterial **(D)** microorganisms at IM across the six Portuguese wine appellations namely, Alentejo, Bairrada, Dão, Douro, Estremadura, and Minho.

The dynamics of microbial communities present at IM, SF, and EF of samples from different Portuguese wine appellations were explored by principal coordinates analysis (PCO; **Figure 4**). For both fungal (**Figure 4A**) and bacterial communities (**Figure 4B**), samples were grouped according to their fermentative stage, where the first axis explains 48 and 52.3% of the total variation, respectively. Interestingly, SF samples were mixed with both IM and EF and, indeed this stage is a transition between IM and EF. As expected, the distribution of the microbial community composition is affected by fermentation. Significant differences (Fungi: *R*_ANOSIM_ = 0.512, *p* = 0.001; Bacteria: *R*_ANOSIM_ = 0.170, *p* = 0.002) between IM, SF, and EF samples were observed for a global test. Conversely, no significant differences were observed between SF and EF samples of the bacterial communities (*R*_ANOSIM_ = 0.155, *p* = 0.954) when analyzed by pairwise tests.

The fungal and bacterial microorganisms responsible for the similarities within each group, and the dissimilarity between the different stages of fermentation, were analyzed using SIMPER analysis (**Supplementary Table S2**). The average of similarity within each group increased over the fermentation process for both fungal (IM: 39.84%; SF: 42.27%; EF: 64.19%) and bacterial community (IM: 42.64%; SF: 48.36%; EF: 46.96%). Further, the fungal communities of IM samples were mainly characterized by the environmental yeasts *Aureobasidium* and *Rhodotorula*, which contributed with 64.55% for the group similarity. Other microorganisms, such as *Hanseniaspora*, *Saccharomyces*, *Lachancea*, *Botryotinia*, *Alternaria*, *Aspergillus*, *Metschnikowia*, *Filobasidiella*, and *Candida* contributed with 25.80% for the group similarity. Regarding the bacterial community at IM, Enterobacteriaceae, Pseudomonadaceae, Microbacteriaceae, Comamonadaceae families contribuited with 52.68% for group similarity, followed by Oxalobacteraceae, Sphingomonadaceae, Xanthomonadaceae, Nocardioidaceae, Methylobacteriaceae, Halomonadaceae, Propionibacteriaceae, Rhodobacteraceae, Micrococcaceae, Acetobacteraceae, which all together contributed with 38.25%.

The analysis of similarity of the fungal community at SF and EF revealed that fewer microorganisms contributed to the similarity of groups when compared with IM, which is explained by the evolution of the fermentative process. In fact, the microbial community tended to be more similar and less diverse at EF. At SF, the microorganisms *Saccharomyces*, *Hanseniaspora*, *Aureobasidium*, and *Lachancea* contibuted with 91.91% for group similarity, and at EF the *Saccharomyces* and *Hanseniaspora* microorganisms contributed with 91.19%. The same behavior was observed for bacterial communities where Enterobacteriaceae, Halomonadaceae, Comamonadaceae, Pseudomonadaceae, and Xanthomonadaceae families contributed with 91.44% of similarity for SF group, whereas Enterobacteriaceae, Comamonadaceae, Acetobacteraceae, Xanthomonadaceae, Pseudomonadaceae, and Oxalobacteraceae families contributed with 91.44% for EF group similarity.

Regarding the comparison between IM, SF, and EF groups of fungal communities, a higher dissimilarity value was obtained for IM vs. EF (86.53%) followed by IM vs. SF (73.84%) and SF vs. EF (53.44%), where microorganisms belonging to the *Lachancea, Saccharomyces, Hanseniaspora, Aureobasidium, Schizosaccharomyces, Candida, Metschnikowia, Torulaspora, Rhodotorula*, and *Alternaria* genera contributed for the dissimilarity of the groups. Furthermore, the diferences of the dissimilary were less pronounced for the bacterial community when compared with fungal population: IM vs EF (66.09%), IM vs SF (66.05%), and SF vs EF (50.51%). Micoorganisms belonging to the Halomonadaceae, Enterobacteriaceae, Pseudomonadaceae, Comamonadaceae, Oxalobacteraceae, Microbacteriaceae, Sphingomonadaceae, Acetobacteraceae, and Xanthomonadaceae familes were those that mostly contributed for the dissimilarity of groups (**Supplementary Table S2**).

### Microbiome of Wine Appellations

In order to understand the biogeographical distribution of microbial populations, the microbiome associated with the six Portuguese appellations was individually compared for IM, SF, and EF, for both bacterial and fungal communities (**Figures 4C,D**). Significant differences were observed across wine appellations for IM samples (Fungi: *R*_ANOSIM_ = 0.305, *p* = 0.003; Bacteria: *R*_ANOSIM_ = 0.321, *p* = 0.014). For both fungal (**Figure 4C**) and bacterial communities (**Figure 4D**), samples were grouped according to their similarity, where the first axis explain 21.5 and 43.4% of the total variation, respectively. The SIMPER analysis (**Supplementary Table S2**) revealed that the average of similarity within each wine appellation was higher at Minho for both bacterial (76.20%) and fungal (63.21%) communities, followed by Estremadura (50.49 and 51.99% for bacterial and fungal populations, respectively), Bairrada (40.81 and 51.77%), Douro (49.68 and 50.68%), Dão (59.74 and 45.29%), and Alentejo (51.54 and 23.98%). The SF samples (fungi: *R*_ANOSIM_ = 0.060, *p* = 0.320; bacteria: *R*_ANOSIM_ = 0.073, *p* = 0.271) and EF samples (fungi: *R*_ANOSIM_ = -0.039, *p* = 0.596; bacteria: *R*_ANOSIM_ = 0.093, *p* = 0.199) did not show any significant differences.

**FIGURE 5:**
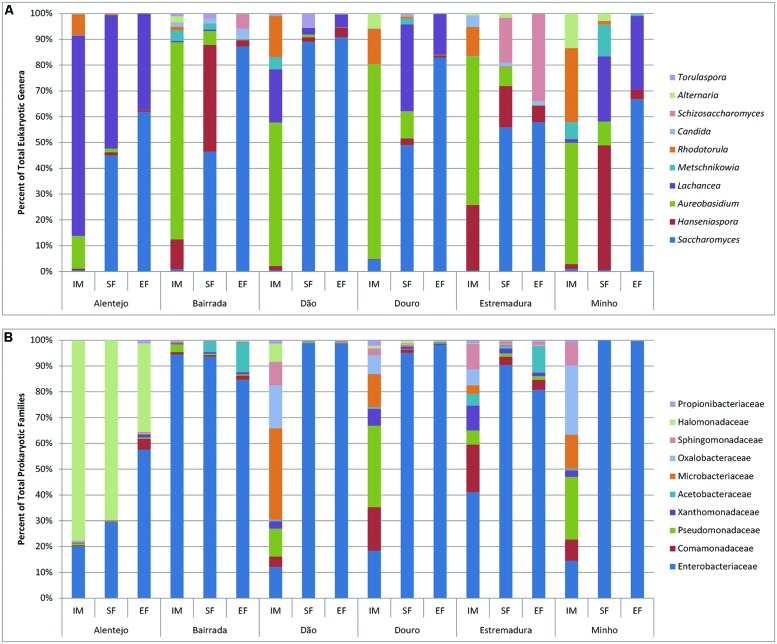
**Eukaryotic **(A)** and prokaryotic **(B)** microbial community distribution over IM, SF and EF of the Portuguese appellations.** Relative abundance of the 10 most abundant eukaryotic **(A)** and prokaryotic **(B)** microorganisms through the genus and family analysis, respectively.

Regarding the fungal microorganisms that contributed for each wine appellation, the genus *Aureobasidium* dominated and contributed for an average of 44.39% appellations similarity (**Supplementary Table S2**). Interestingly, it was observed a regional effect on the contribution of other microorganisms: at Alentejo appellation *Lachancea* prevailed, contributing for 21.44% of region’s similarity; in the Estremadura appellation *Rhodotorula* and *Botryotinia* contributed for 37.96% of the similarity; the Bairrada appellation was characterized by the presence of *Hanseniaspora* and *Ramularia*, who contributed for 18.86% of the regional similarity; the Dão appellation was characterized by the presence of microorganisms from the *Lachancea* and *Rhodotorula* genera (29.07% of similarity); within Douro appellation, *Rhodotorula* and *Erysiphe* contributed with 21.29% for the similarity; and finally, the Minho appellation was characterized by *Rhodotorula* and *Alternaria* (40% of similarity; **Supplementary Table S2**). In general, the fungal populations of IM were characterized by ubiquitous genera as *Aureobasidium, Rhodotorula, Hanseniaspora, Alternaria, Metschnikowia, Saccharomyces, Candida, Ramularia, Penicillium, Lewia, Filobasidiella, Leptosphaerulina*, and *Schizosaccharomyces*, forming the principal structure of the microbial populations (**Figure 5A**).

In SF samples, an increase of *Saccharomyces* population was observed in all regions. Nevertheless, Alentejo had the highest abundance of *Lachancea* and Minho was characterized by having the richest biodiversity, which included *Hanseniaspora, Lachancea, Metschnikowia*, and *Aureobasidium.* Expectedly at EF the dominant genus was *Saccharomyces*, but still some regional differences were observed: samples from Alentejo, Douro, and Minho presented a similar composition (*Saccharomyces* and *Lachancea*), while Bairrada and Dão were mostly composed by *Saccharomyces*. Samples from Estremadura region contained high amounts of both *Saccharomyces* and *Schizosaccharomyces*.

Regarding the bacterial community, the families of Halomonadaceae and Enterobacteriaceae contributed with 91.93% for the Alentejo appellation similarity whereas at Bairrada region, Enterobacteriaceae and Pseudomonadaceae contributed with 75.78%. At Dão appellation, Microbacteriaceae, Oxalobacteraceae, and Enterobacteriaceae contributed with 36.83% and Comamonadaceae, Enterobacteriaceae, Oxalobacteraceae, and Microbacteriaceae families with 52.35% for Douro region similarity. Finally, at Estremadura, Enterobacteriaceae, contributed with 22.47% and at Minho appellation, Oxalobacteraceae, Pseudomonadaceae, and or Enterobacteriaceae with 45.39% for the similarity. It is interesting to notice that the bacterial families responsible for the regional similarities were mostly environmental, and are not related with the oenological process.

In general, the bacterial community was observed to differ across the appellations at IM samples. Additionally, grapes from Alentejo and Bairrada appellations presented the most distinct bacterial profiles (**Figure 5B**). Regarding SF and EF samples, Enterobacteriaceae was ubiquitous to all appellations. Bairrada and Estremadura were also characterized by high amounts of Acetobacteriaceae, while samples from Alentejo presented a unique microbiome characterized by the Halomonadaceae family (**Figure 5B**).

Regarding the most abundant bacterial family, Enterobacteriaceae, microorganisms from the genus *Pantoea* were found in all samples, whereas *Klebsiella* was only detected at IM and SF, and *Tatumella* was only identified at SF and EF samples. Also, bacteria belonging to the Microbacteriaceae family as *Curtobacterium* and *Frigobacterium* were detected in all samples and *Leifsonia* only at IM samples. Concerning all samples, the bacterial genera *Gluconobacter* (Acetobacteraceae) and *Leuconostoc* (Leuconostocaceae) were also abundant, which was expected as they have been long related with wine fermentations. *Variovorax* (Comamonadaceae); *Carnimonas*, *Halotalea*, and *Zymobacter* (Halomonadaceae); *Massilia* (Oxalobacteraceae); *Pseudomonas* (Pseudomonadaceae); and *Sphingomonas* (Sphingomonadaceae) were also extensively detected in all samples.

## Discussion

The aims of this work were to characterize and to compare the diversity of the microbial communities during spontaneous wine fermentations and across different wine Portuguese appellations. To achieve this, high-throughput sequencing was used to fully characterize both eukaryotic and prokaryotic communities from samples collected from six Portuguese wine regions.

Wine fermentations are known to harbor a heterogeneous population of microorganisms. In this work, a diverse set of microbial communities was identified, where the most abundant phyla were Proteobacteria and Ascomycota from prokaryotic and eukaryotic populations, respectively. As expected, a clear relationship was observed between the microbial community and fermentation stage. The biodiversity across the fermentation process decreased for both prokaryotic and eukaryotic communities as a result of the selective environment created over the spontaneous wine fermentation. Interestingly, the variations of biodiversity along this process revealed a higher impact on the fungal community structure, when compared with the bacterial populations. Furthermore, the prokaryotic populations were more diverse than the eukaryotic populations.

In this study, the most abundant eukaryotic microorganisms at IMs were *Aureobasidium* (*A. pullulans*), *Rhodothorula* (*R. nothofagi*), *Hanseniaspora* (*H.uvarum*), and *Lachancea* (*L. thermotolerans*). A diverse set of bacterial population was also uncovered, where Enterobacteriaceae (namely, *Pantoea*, and *Klebsiella*) and Pseudomonadaceae (namely, *Cellvibrio*, and *Pseudomonas*) were the most abundant families. This is in line with the previous reported by Bokulich et al. (2013), where microorganisms as *Cladosporium* spp., *A. pullulans*, *H. uvarum* were detected as the major eukaryotic population in the IMs, and as regards to prokaryotic population, Lactobacillales, Pseudomonadales, or Enterobacteriales were also identified.

The high microbial biodiversity within IM samples was mostly due to environmental microorganisms derived from vineyard. Indeed, several detected microorganisms, namely, *Botryotinia, Phomopsis, Aspergillus*, *Penicillium, Aureobasidium, Rhodotorula*, Enterobacteriaceae, or *Sphingomonas*, were previously described on grapevine leafs and grape surfaces and some of them are even refereed as inhabitant of grapes (Mills et al., 2008; Martins, 2012; Bokulich et al., 2013; Pinto et al., 2014). Also, *Saccharomyces* was detected at IMs, which suggests that this community comes from grapes, reinforcing findings from Bokulich et al. (2013), Pinto et al. (2014), and Taylor et al., 2014.

Regarding the origin of spoilage microorganisms, there has been a vivid discussion on whether or not these are present at the vineyards, where grapes are the principal source for wine contamination and deterioration (Renouf et al., 2005), or otherwise, winemaking equipment is the source of spoilage microorganisms (Couto et al., 2005). For instance, it is considered that *Dekkera*/*Brettanomyces*, the lactic and AAB are the most important wine spoilage microorganisms (Bartowsky et al., 2003; Beneduce et al., 2004; Cocolin et al., 2004). In this study, *Dekkera*/*Brettanomyces bruxellensis* was not detected, which is in line with the study of Suárez et al. (2007), who reported that this spoilage yeast is mainly present in winemaking equipment with deficient cleaning; and is opposed to the findings reported by Renouf and Lonvaud-Funel (2007). Still, these results *per se* do not yet allow for a clear conclusion on their origin. In the other hand, LAB and AAB were detected at low abundances, but *Oenococcus oeni*, a LAB extensively used to carry out the MLF, was not detected. Additionally, filamentous fungi (molds) were identified on IMs: *Alternaria, Aspergillus, Botrytis, Cladosporium, Penicillium*, or *Rhizopus*, which are undesirable for wine quality (Toit and Pretorius, 2000). *Aspergillus* (*A. niger*) and *Penicillium* (*P. glabrum* and *P. brevicompactum*) were found in all the appellations considered in this work. However, and along fermentations, these molds disappeared, which supports the observations that they are sensitive to the wine fermentation conditions (Blesa et al., 2006).

From the IM to the wine, sequential stages of microbial development were observed, as result of fermentation activities (Fleet et al., 1984; Jolly et al., 2003). An initial growth of non-*Saccharomyces*, such as *Hanseniaspora, Torulaspora*, *Metschnikowia*, and *Pichia* at SF was followed by a decrease or even a disappearance of these yeasts at the EF and, conversely, the increase of *S. cerevisiae* was evidenced. A similar kinetic pattern was also observed on prokaryotic community, where in transition from IM to SF, Enterobacteriaceae family increased, and then decreased from SF to EF, specifically in Bairrada, Dão, and Estremadura appellations.

In spontaneous wine fermentations, *S. cerevisiae* was dominant despite the high abundance of *Hanseniaspora* and *Lachancea*. Yeasts associated with wine fermentation such as *Metschnikowia* (*M. pulcherrima and M. viticola*), *Torulaspora* (*T. delbrueckii*), *Schizosaccharomyces* (*S. japonicus*), *Candida* (*C. zemplinina*), *Issatchenkia* (*I. terricola*), and, less frequently, *Pichia* (*P. kluyveri* and *P. kudriavzevii*) were also detected. However, their relative abundances varied according to their appellation of origin. Indeed, each appellation presented characteristic microbial communities, with different abundances of non-*Saccharomyces* and specific patterns of microbial communities. Interestingly, *Schizosaccharomyces* (*S. japonicus*) was also detected, even at later stages, and was present at higher abundances in the Estremadura region. This yeast is characterized by having a high fermentative capacity at high temperatures (optimal growth around 30°C), and by being resistant to SO_2_ and to the stringent conditions of fermentation (Torija et al., 2001). Regarding *Torulaspora delbrueckii*, it was found until EF, and it has been previously reported to survive until later stages of fermentation and to produce lower levels of acetic acid (Ciani et al., 2006). Interestingly, samples which presented higher abundance of this microorganism also generally had higher abundance of AAB namely, *Gluconobacter* (*G. oxydans*).

Among bacterial communities, during the fermentation, Enterobacteriaceae was the most abundant family (namely, *Tatumella* sp.). Nisiotou et al. (2011) also showed that Enterobacteriaceae persists in fermentation, and Ruiz et al. (2010) also confirmed its prevalence at beginning, mid and final stages of MLFs in different Spanish wineries. This raises the question if these bacteria interact with fermenting yeasts and, if so, in what degree can this microbial population influence (negatively or positively) the organoleptic proprieties of wine. The bacterial populations were found to be less dynamic than the eukaryotic populations in the later stages of fermentation process, and their geographic profiles were more similar: it was observed a clear dominance of Enterobactereaceae family at all appellations but Alentejo, where microorganisms from Halomonadaceae family were also presented with high abundance. The Bairrada and Estremadura appellations were also characterized by the presence of microorganisms from the Acetobacteraceae family. Among the LAB, high amounts of *Lactobacillus* (Lactobacillaceae), *Leuconostoc* (Leuconostocaceae), *Lactococcus*, and *Streptococcus* (Streptococcaceae) were detected. Additionally, *Facklamia* (Aerococcaceae), *Carnobacterium, Dolosigranulum, Granulicatella*, and *Trichococcus* from Carnobacteriaceae family, *Enterococcus* (Enterococcaceae) and *Weisella* as *W. cibaria* (Leuconostocaceae) were also detected, but at lower abundances. Interestingly, and with exception of *Weisella*, those specific microorganisms had not been previously isolated from musts and wines (König and Fröhlich, 2009).

To investigate whether or not there is a geographic imprint on the wine fermentation microbiome, a PCO was performed for each fermentation stage in order to evaluate differences according to wine appellation. Interestingly, significant differences (*p* < 0.05) were observed for both fungal and bacterial microbial communities at IM between wine appellations. These results are consistent with those reported by Bokulich et al. (2013), who observed differences in the microbial community structure across wine appellations from California. Over the fermentation process, the initial microbiome associated with each wine appellation disappears and, as a consequence, the biogeographic profile was lost (no significant differences were observed for SF and EF). As observed, this microbiome is characterized by the presence of environmental microorganisms, which constituted a signature of each Portuguese wine regions. Moreover, these results also suggested that the initial microbial community could strongly contribute to the uniqueness of the wines derived from each specific wine appellation. Furthermore, each wine appellation presented its own pattern of biodiversity that varied in terms of the microbial abundance. This finding is of special interest when considering the non-saccharomyces population at the SF, whom have been acknowledged for their metabolic contribution to the final wine sensorial properties (Romano et al., 2003; Jolly et al., 2014), which reinforces their role on the regional attributes of wines. These findings open new horizons to dissect how microbiomes affect wine properties and support the need to unveil the endogenous microflora of such regions and explore its natural microbial populations in order to produce valuable wines styles.

## Conflict of Interest Statement

The authors declare that the research was conducted in the absence of any commercial or financial relationships that could be construed as a potential conflict of interest.

## Supplementary Material

**Figure S1 | Portugal map with the appellations and grape varieties chosen for study**. Sample collection was done in 6 appellations – Minho (M), Douro (Dr), Dão (D), Bairrada (B), Estremadura (E), and Alentejo (Al). The grave varieties collected were Al, Alvarinho; Ax, Alfrocheiro; B, Baga; J, Jean; L, Loureiro; P, Piriquita; T, Trincadeira; TF, Touriga Franca; TN, Touriga Nacional; and TR, Tinta Roriz (also known Aragonez).

**Figure S2 | Rarefaction curves at a genetic distance of 3% for each sample (IM, SF, and EF)**. D2 **(A)** and ITS2 **(B)** sequences both from the analysis of 26S rRNA and ITS regions of eukaryotic population present in the sample and V6 sequences **(C)** from the analysis of 16S rRNA of prokaryotic diversity. The IM, start fermentation (SF) and end of fermentation (EF) are represented by the blue, yellow and green color, respectively.

**Table S1 | Total sequences obtained for eukaryotic (ITS2 and D2) and prokaryotic (V6) microbial communities for IM, SF, and EF from different wine appellations**. Operational taxonomic units (OTUs) and estimated species (Chao1) were determined at a genetic distance of 3% using Mothur. The coverage obtained was also determined as being the ratio between the observed OTUs and the estimated Chao1 (OTUs/Chao1).

**Table S2 | Analysis of the similarity and dissimilarity across wine fermentation stages and wine appellations**. The similarity and dissimilarity across wine fermentation stages namely, initial musts (IM), start of fermentation (SF), and end of fermentation (EF) and wine appellations were calculated through the SIMPER analysis.
