# Supplementary figures and images for: Wine fermentation microbiome: a landscape from different Portuguese wine appellations
Source: Front Microbiol. 2015 Sep 1;6:905. doi: 10.3389/fmicb.2015.00905 (PMC4555975; doi:10.3389/fmicb.2015.00905)

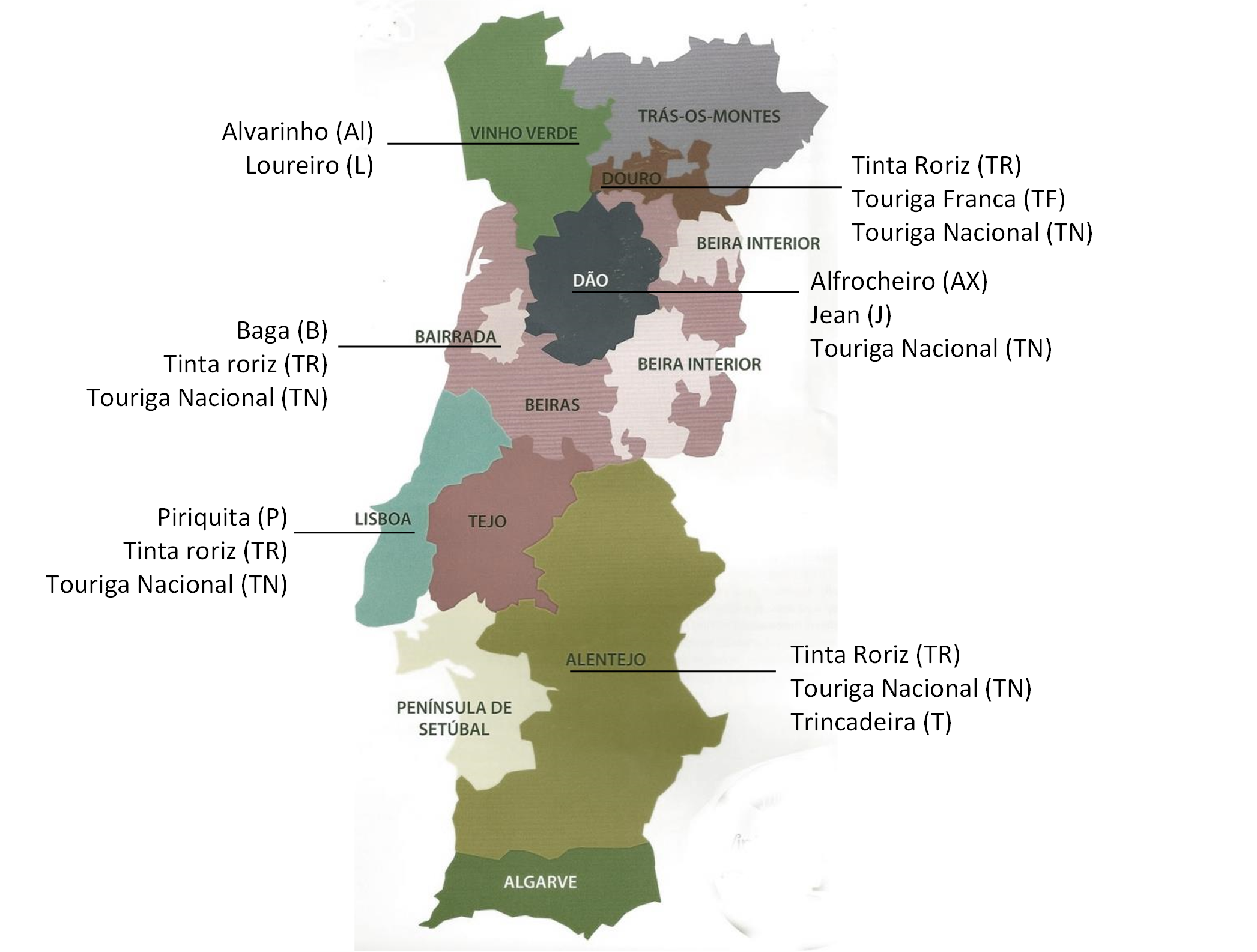

Supplement: Supplementary file 1 [file Image_1.TIFF]

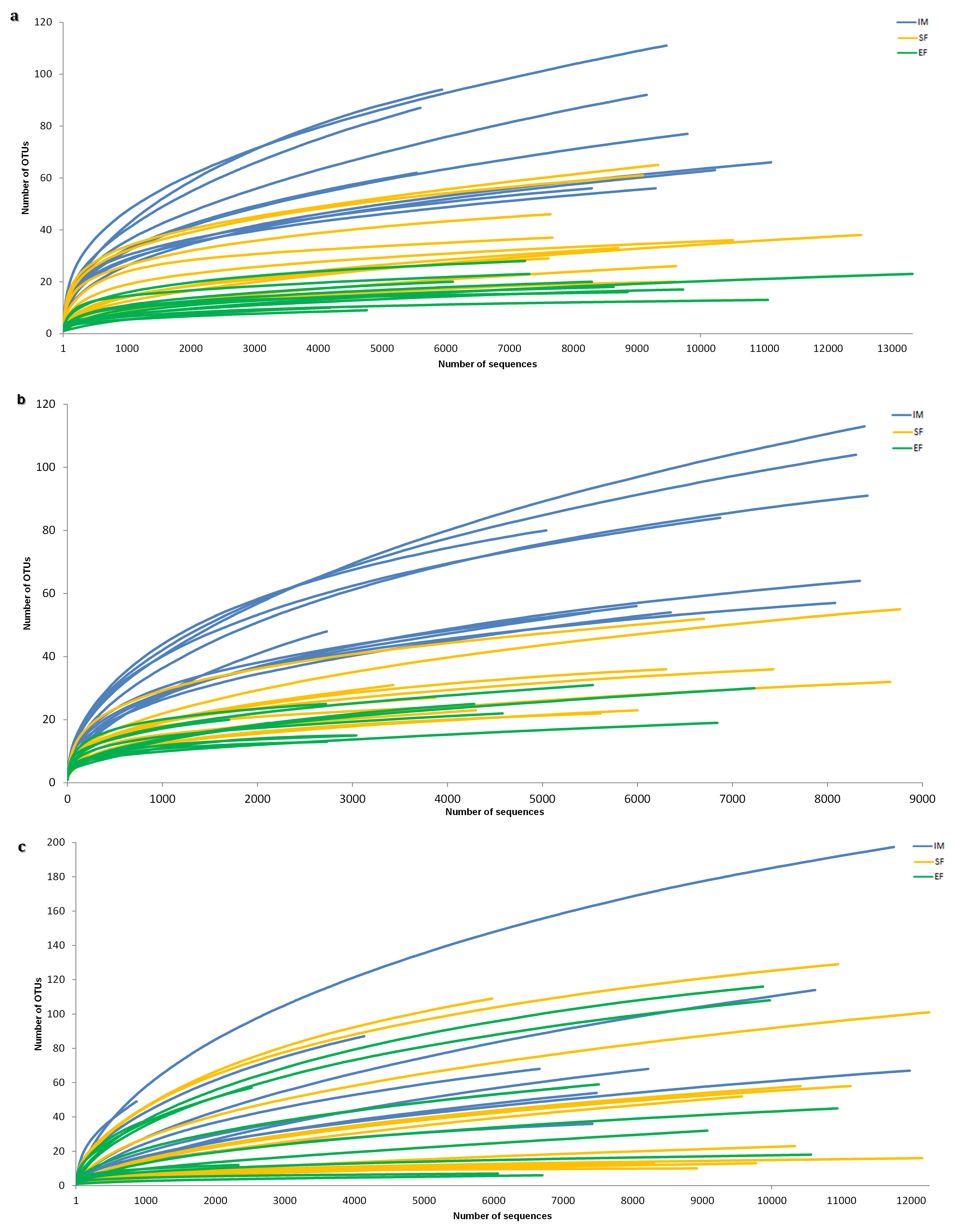

Supplement: Supplementary file 2 [file Image_2.TIF]
